# Supplementary figures and images for: Infection increases activity via Toll dependent and independent mechanisms in Drosophila melanogaster
Source: PLoS Pathog. 2022 Sep 21;18(9):e1010826. doi: 10.1371/journal.ppat.1010826 (PMC9529128; doi:10.1371/journal.ppat.1010826)

A

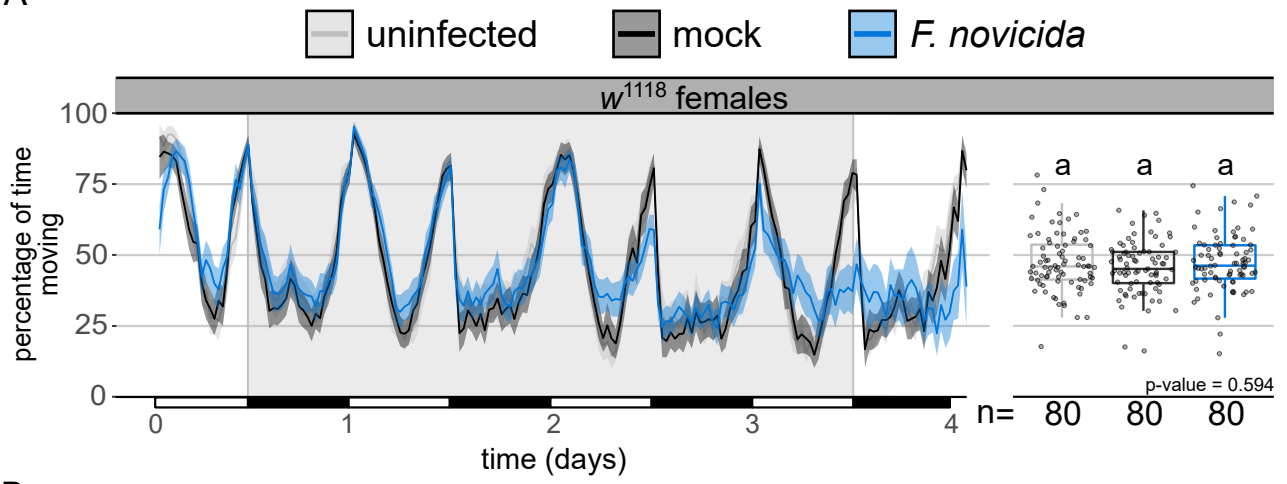

B

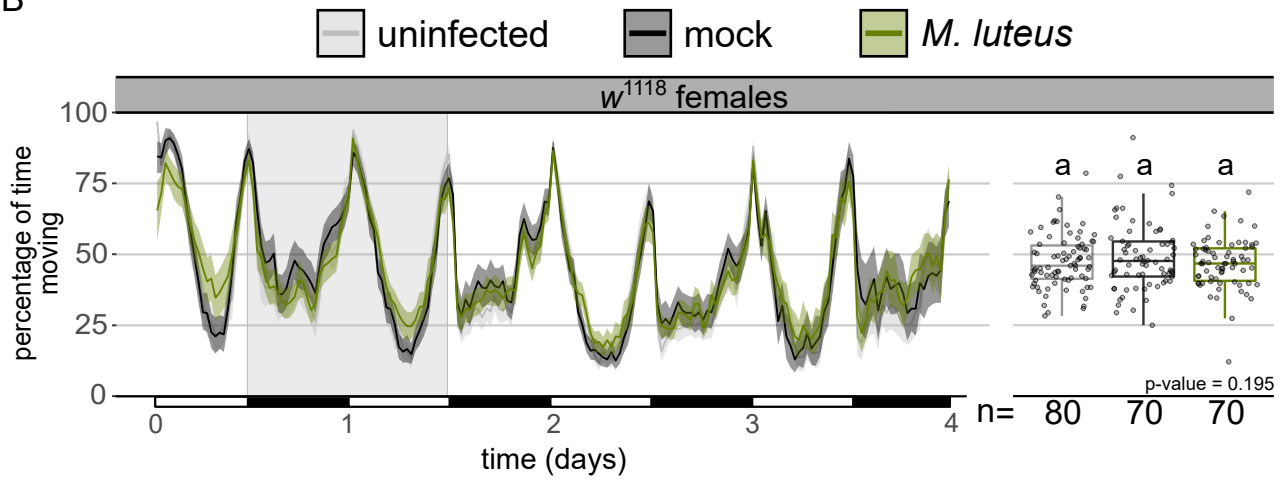

Supplement: S1 Fig — Ethogram showing percentage of time wild-type female flies spend moving over time in 30-min bins during infection with (A) Francisella novicida or (B) Micrococcus luteus (F. novicida: Kruskal-Wallis chi-square = 1.042, df = 2, n = 240, p = 0.594; M. luteus: Kruskal-Wallis chi-square = 3.263, df = 2, n = 220, p = 0.196). (PDF) [file ppat.1010826.s001.pdf]

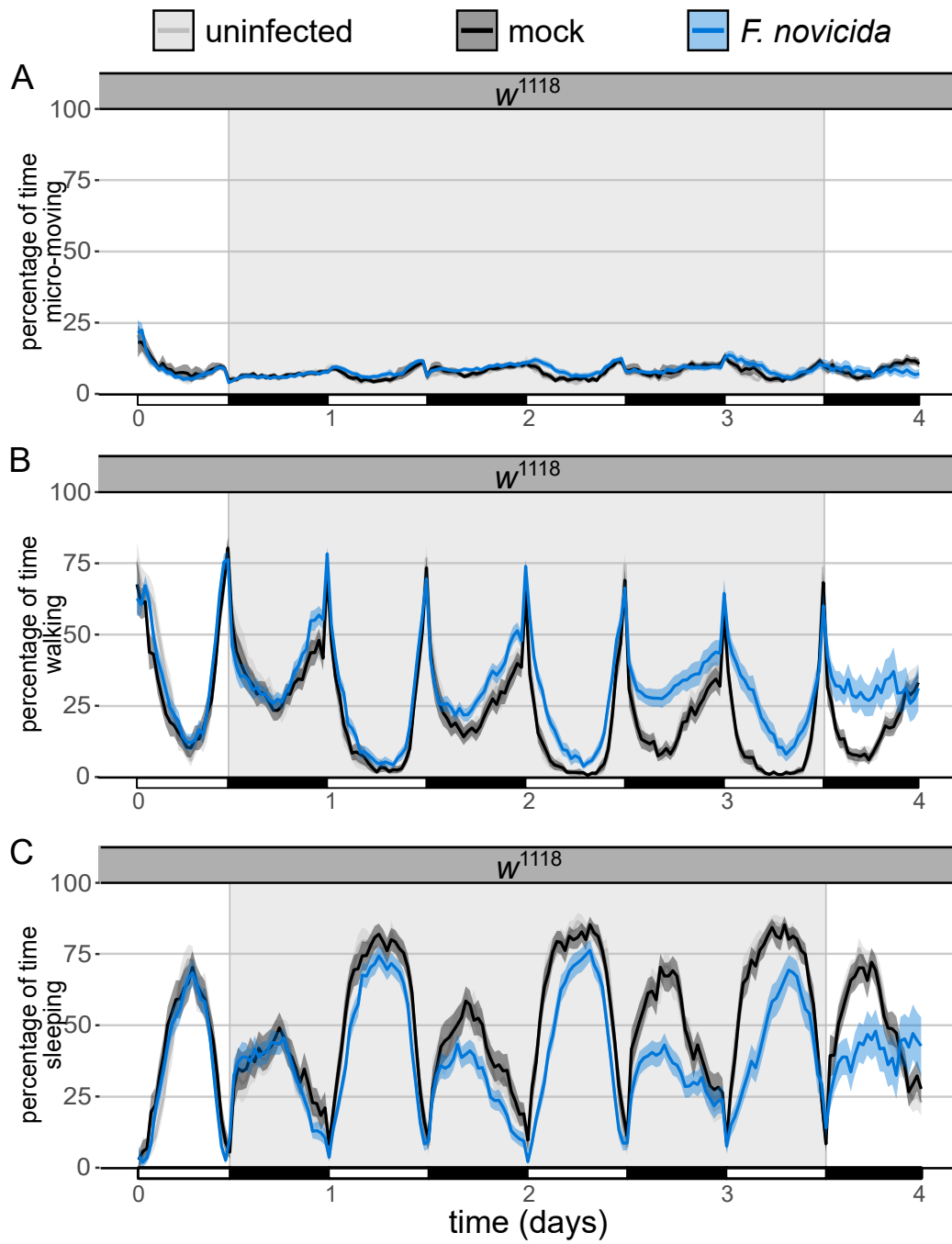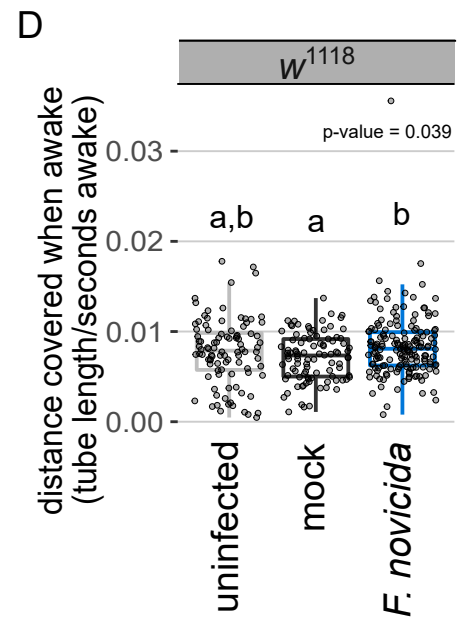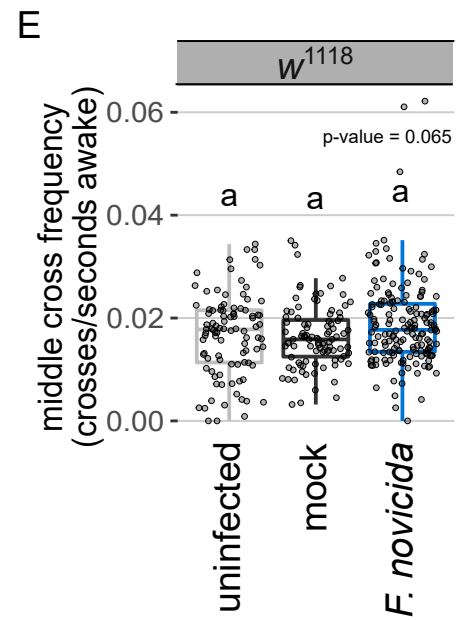

Supplement: S2 Fig — Ethogram showing percentage of time infected wild-type males spend (A) engaging in micromovements (e.g. feeding and grooming), (B) walking and (C) sleeping in 30-min bins. Boxplots show the quantification of (D) total distance covered when flies were scored as awake and (E) the total number of times that the flies crossed the middle of the housing tube as a proportion of the time (in seconds) spent awake. Uninfected and mock controls are represented by grey and black tracings, respectively. Infected flies are in blue. Distance covered normalized to time awake (Kruskal-Wallis chi-square = 6.496, df = 2, n = 419, p = 0.039; Dunn’s post hoc: mock|F. novicida = 0.033, mock |uninfected = 0.163, uninfected|F. novicida = 0.460) and midline crosses normalized to time awake (Kruskal-Wallis chi-square = 5.453, df = 2, n = 419, p = 0.065; Dunn’s post hoc: mock|F. novicida = 0.064, mock |uninfected = 0.330, uninfected|F. novicida = 0.339) were not impacted by the infection. (PDF) [file ppat.1010826.s002.pdf]

**A**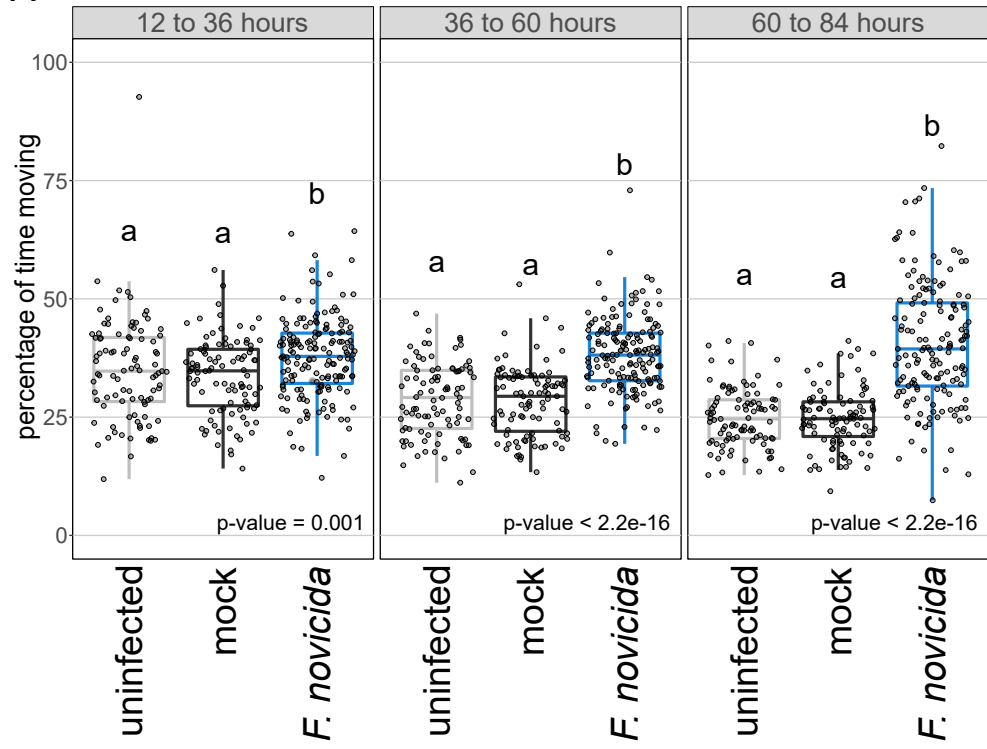**B**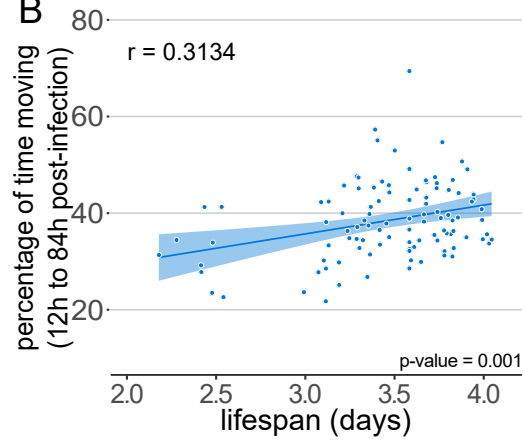**C**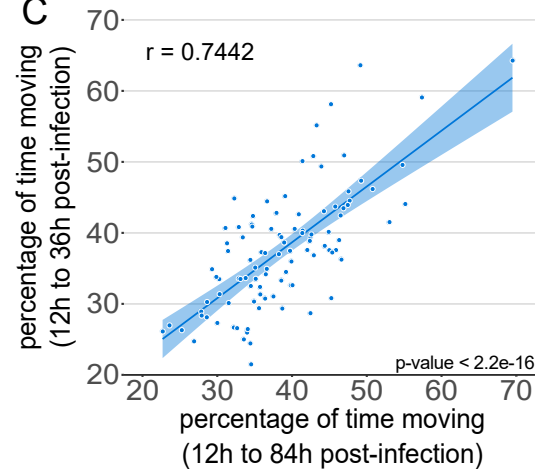

Supplement: S3 Fig — (A) Boxplots showing day-by-day activity of infected wild-type males. 12-36h: (Kruskal-Wallis chi-square = 14.085, df = 2, n = 419, p = 8.7e-04; Dunn’s post hoc: mock|F. novicida = 0.001, mock |uninfected = 0.346, uninfected|F. novicida = 0.02). 36-60h: (Kruskal-Wallis chi-square = 98.325, df = 2, n = 419, p = 2.2e-16; Dunn’s post hoc: mock|F. novicida = 3.1e-17, mock |uninfected = 0.519, uninfected|F. novicida = 7.9e-15). 60-84h: (Kruskal-Wallis chi-square = 137.88, df = 2, n = 419, p = 2.2e-16; Dunn’s post hoc: mock|F. novicida = 1.3e-22, mock |uninfected = 0.869, uninfected|F. novicida = 9.9e-22). (B) Activity level throughout infection was not correlated with survival (Pearson’s correlation, r = 0.313; t = 3.31, df = 101, p = 0.001) and (C) activity levels on day 1 are positively correlated with total activity (Pearson’s correlation, r = 0.744; t = 11.2, df = 101, p = 2.2e-16). Data from multiple replicates are shown. (PDF) [file ppat.1010826.s003.pdf]

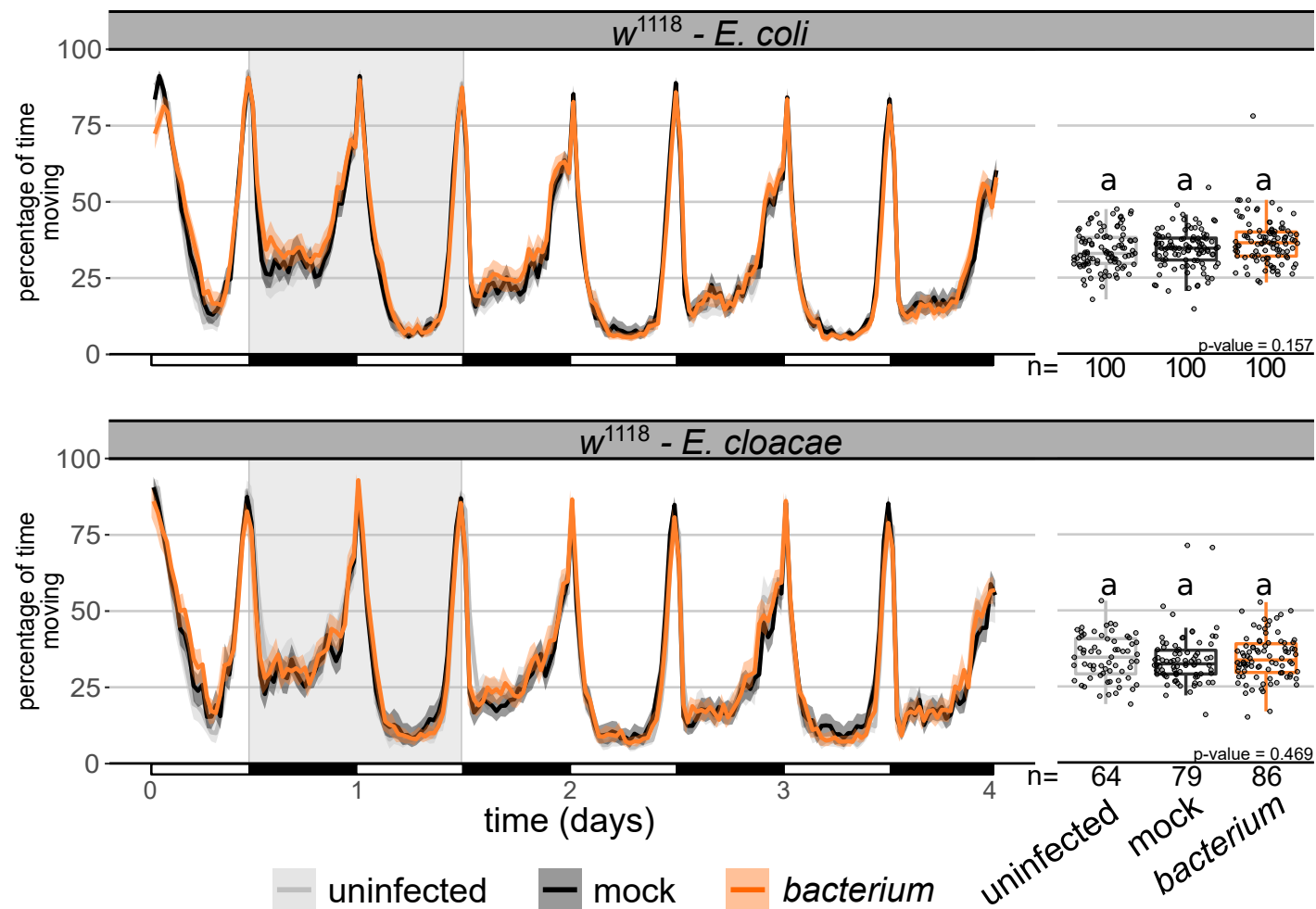

Supplement: S4 Fig — Ethograms showing percentage of time flies spend moving over time in 30-min bins. Uninfected and mock controls are represented by grey and black tracings, respectively. Infected flies are in orange. Neither of these extracellular/Gram-negative bacteria, Escherichia coli and Enterobacter cloacae induced activity (E. coli: Kruskal-Wallis chi-square = 3.699, df = 2, n = 300, p = 0.16; E. cloacae: Kruskal-Wallis chi-square = 1.516, df = 2, n = 229, p = 0.47). Data from multiple replicates are shown. (PDF) [file ppat.1010826.s004.pdf]

uninfected    mock    *F. novicida*    *M. luteus*

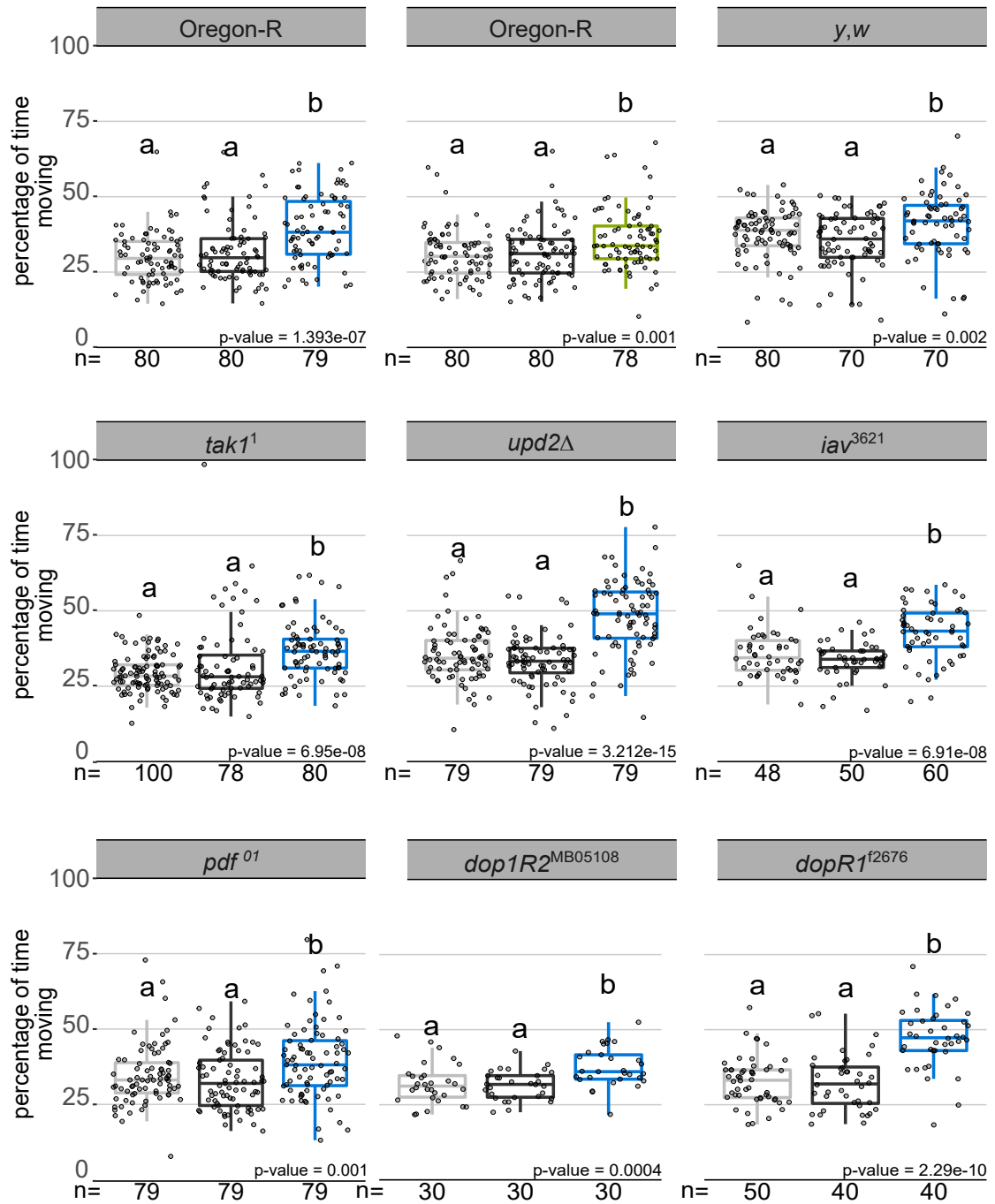

Supplement: S5 Fig — Boxplots showing the percentage of time flies spend moving over time. Uninfected and mock controls are represented by grey and black tracings, respectively. Infected flies are in blue. Previously characterized phenotypes and statistics of studied mutants can be found in S1 and S2 Tables. Data from multiple replicates are shown. (PDF) [file ppat.1010826.s005.pdf]

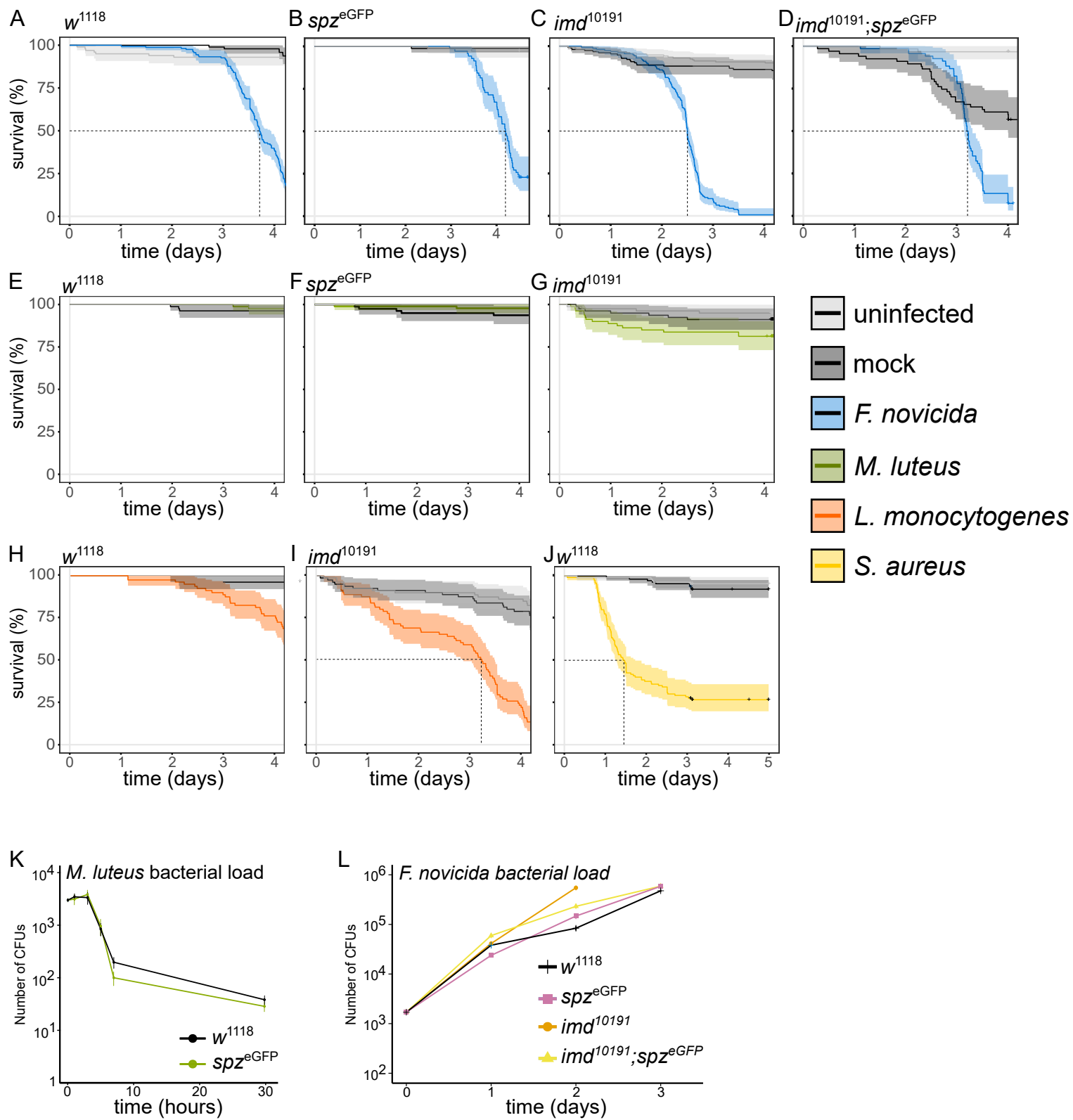

Supplement: S6 Fig — (A–J) In all plots, grey and black tracings represent uninfected and mock controls, respectively. F. novicida, Micrococcus luteus, Listeria monocytogenes and Staphylococcus aureus infections are shown in blue, green, orange and yellow, respectively. Francisella novicida infection was lethal in all four genotypes. Infection with M. luteus did not result in more lethality than either uninfected or mock controls, while L. monocytogenes and S. aureus both lead to decreased survival. Median survival is indicated by dotted lines intersecting the y and x axes at 50% survival and time (in days), respectively. Survival was calculated at the same time as activity data and thus have the same sample size as indicated elsewhere. Data from multiple replicates are shown. (K) Quantification of M. luteus markers represent means and whiskers represent SE. Initial inoculum consisted of ~ 5000 colony forming units (CFUs). Within 30h bacterial numbers decreased to near-undetectable levels (average of 28–40 CFUs/fly). (L) Quantification of F. novicida. Bacterial numbers increase over the course of infection. All genotypes were injected with the same initial dose (t = 0; ~1700 CFUs). The last measured timepoint was 24h prior to the onset of death for each genotype; this was 72h for all genotypes except imd10191 which was 48h. Genotypes are represented by marker style and line colour as indicated inset. Markers indicate means and whiskers represent SE. Bacterial quantifications were repeated at least twice, n = 16–22 flies/genotype/timepoint; data from all replicates are shown. (PDF) [file ppat.1010826.s006.pdf]

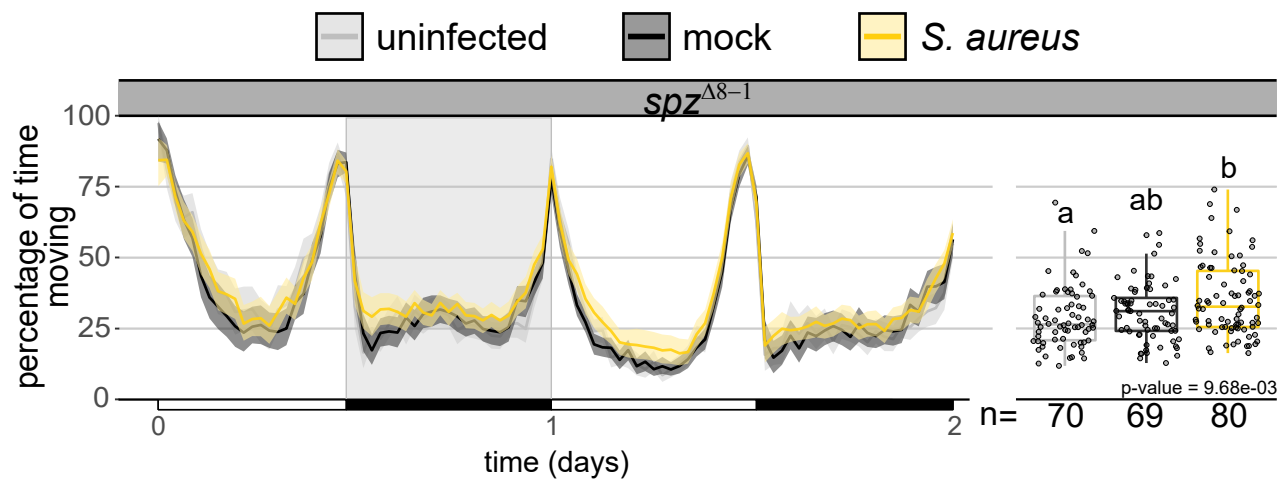

Supplement: S7 Fig — Ethogram showing percentage of time spz Δ8–1 mutant flies spend moving over time in 30-min bins after infection with Staphylococcus aureus (Kruskal-Wallis chi-square = 9.276, df = 2, n = 219, p = 9.68e-03; Dunn’s post hoc: mock|S. aureus = 0.121, mock|uninfected = 0.221, uninfected|S. aureus = 7.56e-03). (PDF) [file ppat.1010826.s007.pdf]

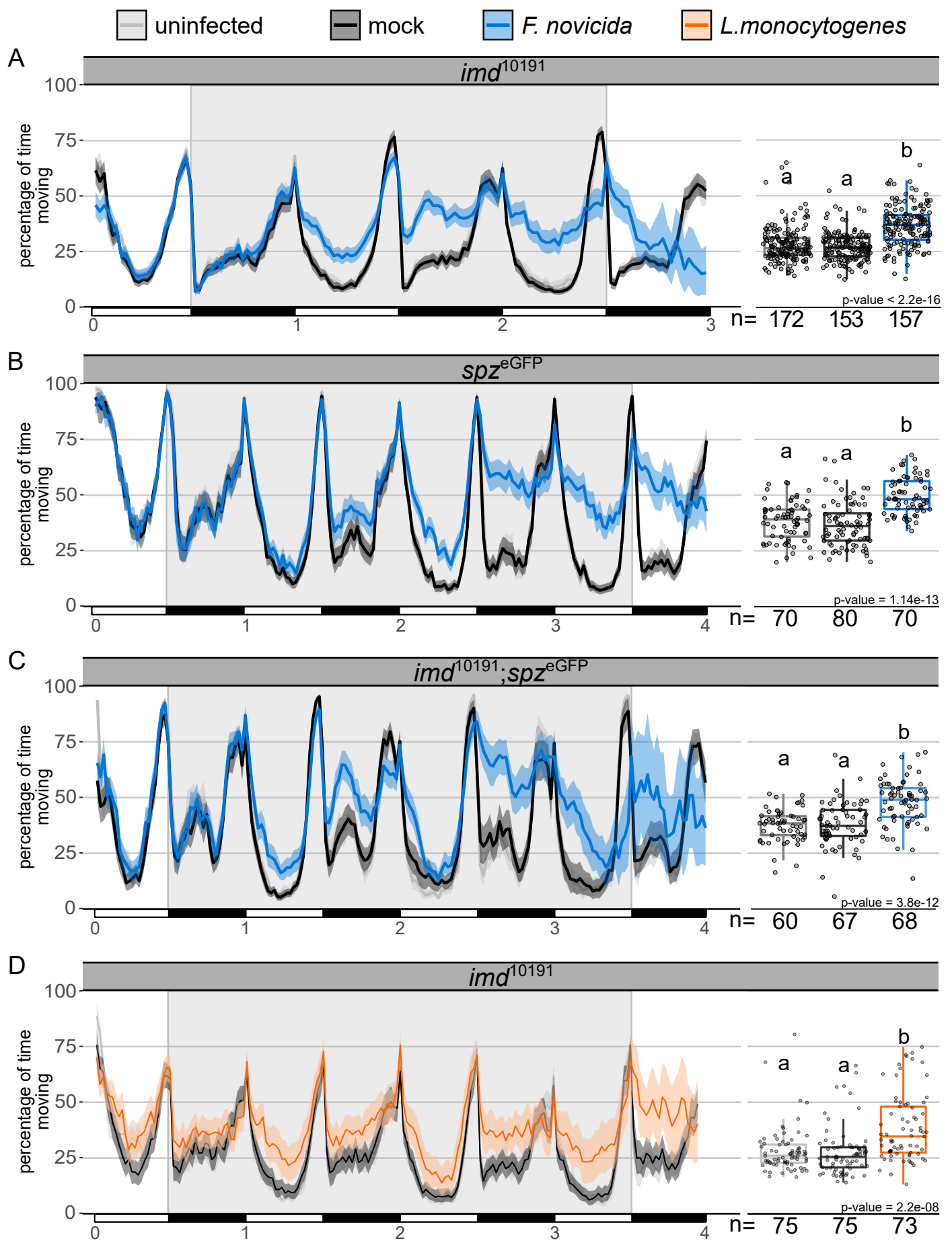

Supplement: S8 Fig — Ethogram showing percentage of time (A) imd10191 (B) spzeGFP and (C) imd10191; spzeGFP, flies spend moving in 30-min bins during Francisella novicida infection. Infected animals moved significantly more than both the uninfected and mock controls (imd: Kruskal-Wallis chi-square = 111.32, df = 2, n = 482, p = 2.2e-16; Dunn’s post hoc: mock|F. novicida = 1.3e-20, mock|uninfected = 0.38, uninfected|F. novicida = 1.4e-18; spzeGFP: Kruskal-Wallis chi-square = 59.59, df = 2, n = 220, p = 1.1e-13; Dunn’s post hoc: mock|F. novicida = 1.7e-12, mock|uninfected = 0.36, uninfected|F. novicida = 1.6e-09; imd;spzeGFP: Kruskal-Wallis chi-square = 52.594, df = 2, n = 195, p = 3.8e-12; Dunn’s post hoc: mock|F. novicida = 5.6e-09, mock|uninfected = 0.45, uninfected|F. novicida = 1.9e-10). Data from multiple replicates are shown. (D) imd10191 mutant flies exhibit increased activity during infection with Listeria monocytogenes (Kruskal-Wallis chi-square = 35.306, df = 2, n = 223, p = 2.16e-08; Dunn’s post hoc: mock|L. monocytogenes = 1.22e-07, mock|uninfected = 0.454, uninfected| L. monocytogenes = 3.13e-06). (PDF) [file ppat.1010826.s008.pdf]

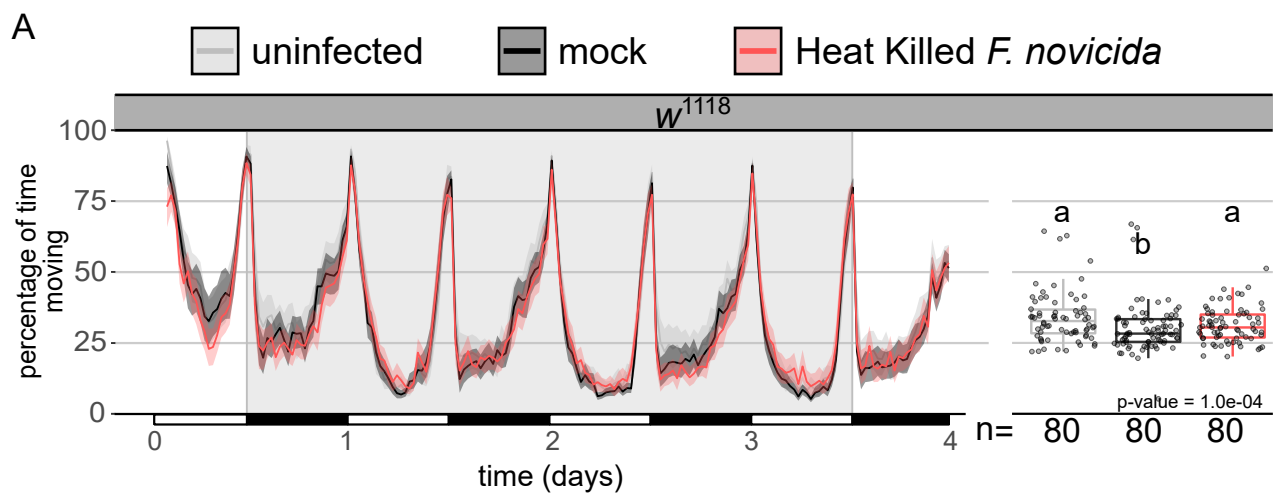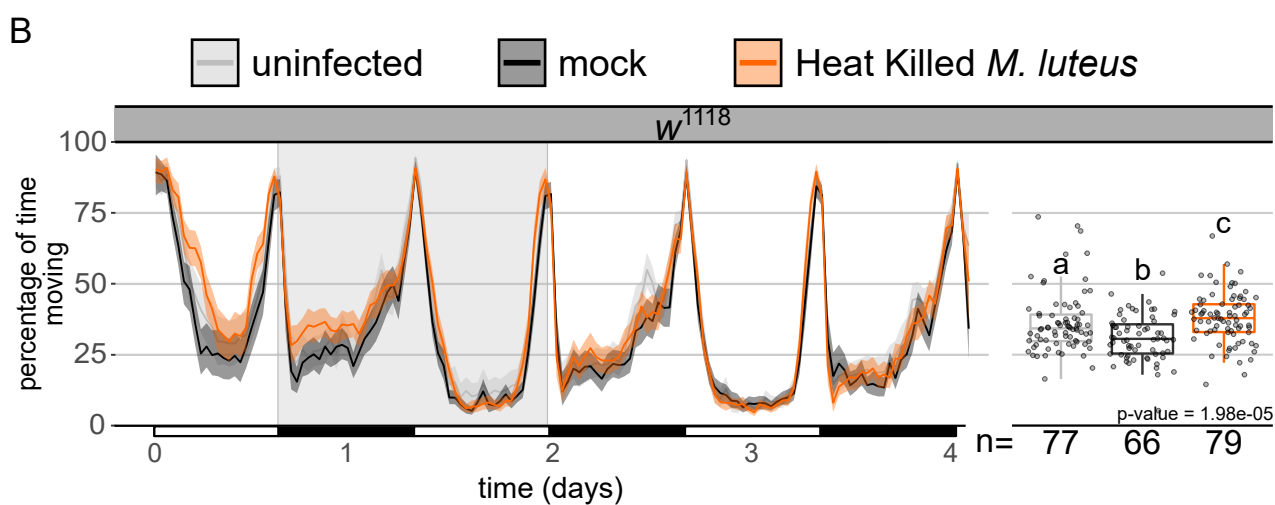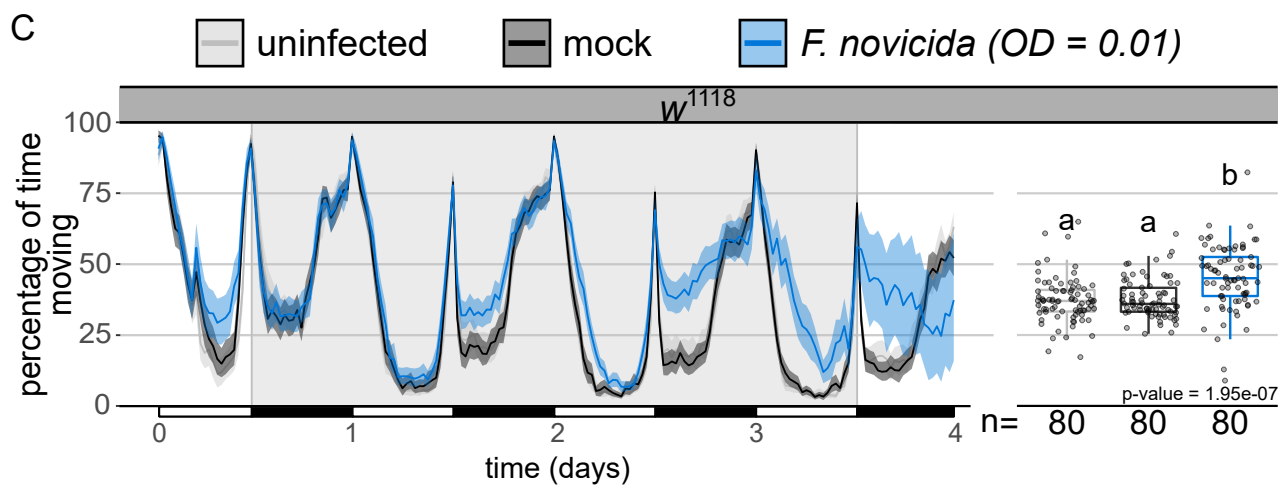

Supplement: S9 Fig — Ethogram showing percentage of time wild-type flies spend moving over time in 30-min bins during infection with (A) heat killed Francisella novicida, (B) heat killed Micrococcus luteus or (C) a low dose (~170 CFUs) of F. novicida. Injection with heat-killed F. novicida did not result in a significant increase in activity (Kruskal-Wallis chi-square = 18.40, df = 2, n = 240, p = 1e-04; Dunn’s post hoc: mock|HK F. novicida = 0.011, mock|uninfected = 6.70e-05, uninfected|HK F. novicida = 0.120). Flies injected with heat-killed M. luteus moved significantly more than both the uninfected and mock controls (Kruskal-Wallis chi-square = 21.66, df = 2, n = 222, p = 1.98e-05; Dunn’s post hoc: mock|HK M. luteus = 9.89e-06, mock|uninfected = 1.09e-02, uninfected|HK M. luteus = 4.16e-02). Infection with low dose of F. novicida led to increased activity (Kruskal-Wallis chi-square = 37.315, df = 2, n = 240, p = 7.89e-09; Dunn’s post hoc: mock|low-F. novicida = 1.95e-07, mock|uninfected = 0.991, uninfected| low- F. novicida = 3.05e-07). (PDF) [file ppat.1010826.s009.pdf]

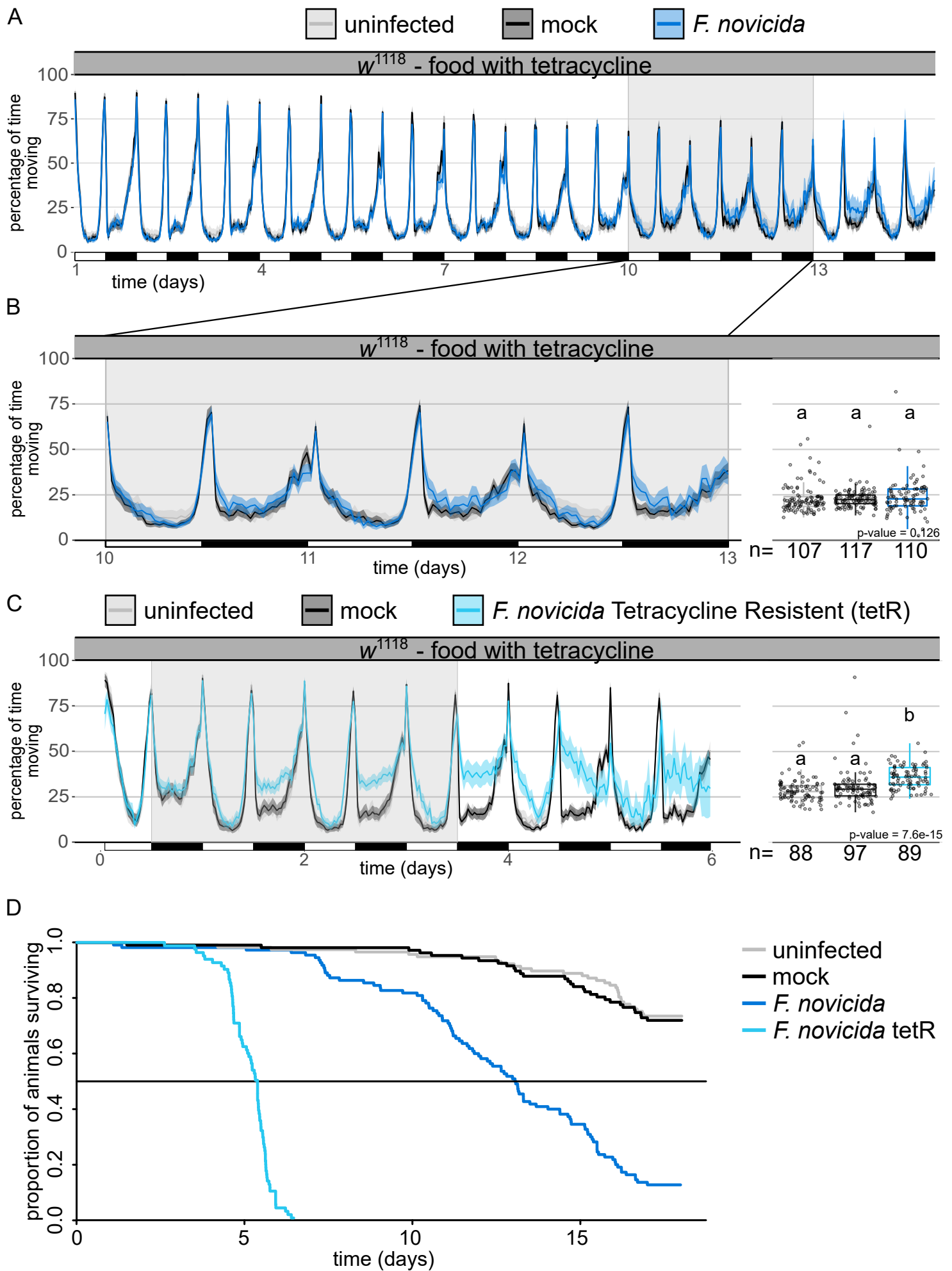

Supplement: S10 Fig — Ethogram showing percentage of time wild-type male flies spend moving over time in 30-min bins during infection with (A) wild-type Francisella novicida treated with the antibiotic tetracycline, (B) shows an inset of (A) highlighting days 10–12 after infection (Kruskal-Wallis chi-square = 4.138, df = 2, n = 334, p = 0.126). (C) Ethogram for wild-type flies infected with a tetracycline-resistant strain of F. novicida under tetracycline treatment (Kruskal-Wallis chi-square = 65.011, df = 2, n = 274, p = 7.64e-15; Dunn’s post hoc: mock|tet-R F. novicida = 2.18e-10, mock|uninfected = 0.196, uninfected| tet-R F. novicida = 1.88e-13). (D) Survival plot for flies treated with tetracycline and infected with wild-type and tetR F. novicida. (PDF) [file ppat.1010826.s010.pdf]

FED STARVED

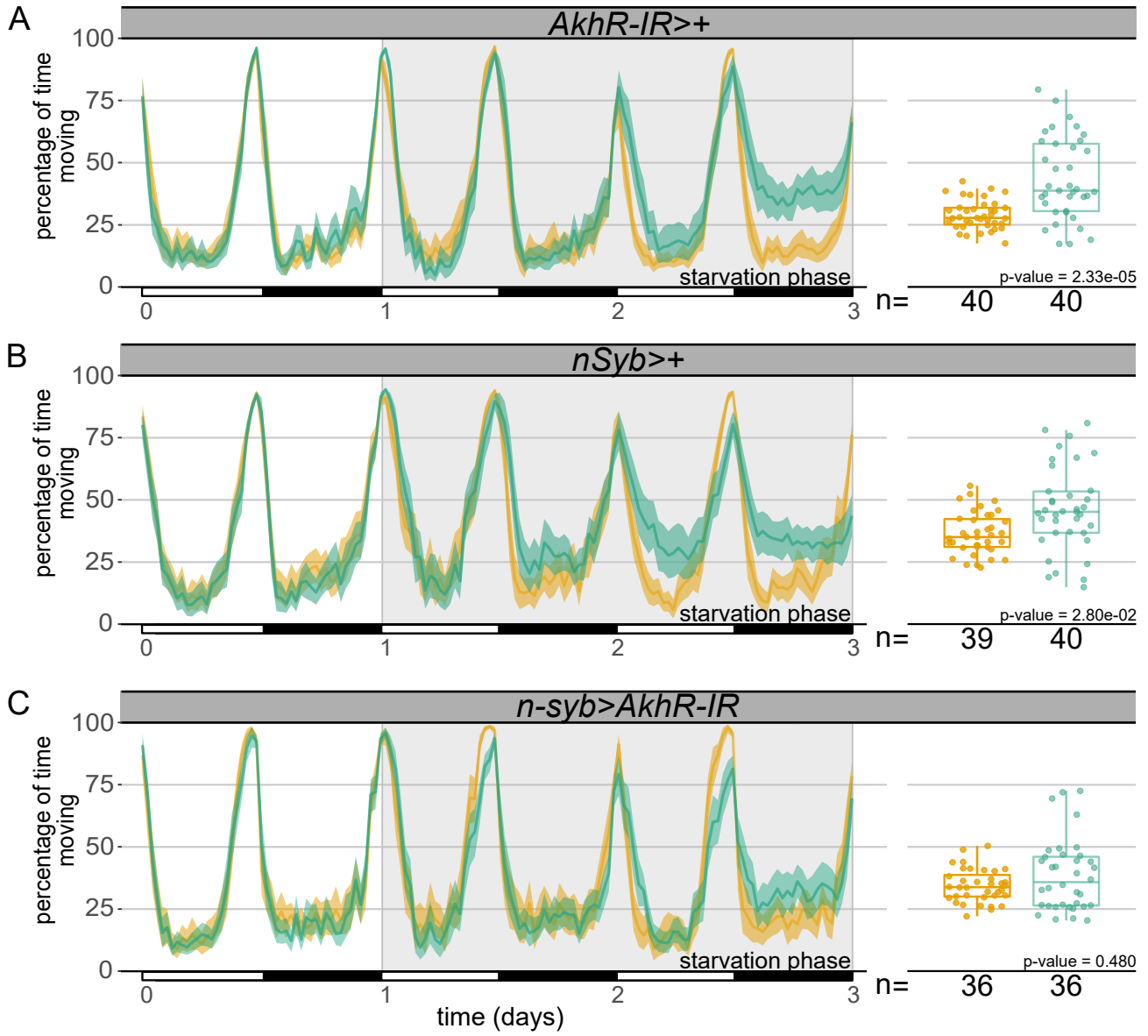

Supplement: S11 Fig — Ethogram showing percentage of time (A) AkhR-IR>+, (B) n-sybG4>+, and (C) n-sybG4/UAS—AKH-R RNAi male flies spend moving over time in 30-min bins. The grey area indicates the starvation period, when flies were transferred from standard food to starvation food (2% agarose in PBS). Boxplots indicate the quantification of the entire starvation period (Kruskal-Wallis chi-square = 39.088, df = 5, n = 231, p = 2.28e-07; Dunn’s post hoc: UAS—AKH-R RNAi>+ FED|STARVED = 2.33e-05, n-sybG4>+ FED|STARVED = 2.80e-02, n-syb>AKH-R RNAi = 0.480). All genotypes were assayed at the same time but are here shown separately for clarity. Experiments conducted at 25°C. (PDF) [file ppat.1010826.s011.pdf]
