# Supplementary material for: Infection increases activity via Toll dependent and independent mechanisms in Drosophila melanogaster
Source: PLoS Pathog. 2022 Sep 21;18(9):e1010826. doi: 10.1371/journal.ppat.1010826 (PMC9529128; doi:10.1371/journal.ppat.1010826)
Supplement: S1 Table — Reported phenotypes for the tested activity mutants associated with immune response or general physical activity levels. (DOCX) [file ppat.1010826.s012.docx]

**S1 Table. Phenotypes of activity mutants tested.**

| Gene | Published phenotype |
| --- | --- |
| yw | Alternative ‘control’ line; abnormal colour (yellow) & male courtship behaviour |
| *tak1*^1^ | Highly susceptible to Gram negative bacteria |
| *upd2^∆^* | Defective immune response |
| *pdf*^01^ | Locomotor & circadian behaviour defective |
| *dop1R2*^MB05108^ | Hypoactive |
| *dopR1*^f2676^ | Hyperactive |
| *iav*^3621^ | Locomotor behaviour defective |
