# Supplementary material for: Infection increases activity via Toll dependent and independent mechanisms in Drosophila melanogaster
Source: PLoS Pathog. 2022 Sep 21;18(9):e1010826. doi: 10.1371/journal.ppat.1010826 (PMC9529128; doi:10.1371/journal.ppat.1010826)
Supplement: S2 Table — Statistics for differences in activity levels between infection conditions for the different mutants tested. (DOCX) [file ppat.1010826.s013.docx]

**S2 Table. Statistics from activity mutant assays.**

| **Genotype** | **Kruskal-Wallis Test Statistics** | **Uninfected\|PBS** | **Uninfected\|*F. novicida*** | **PBS\|*F. novicida*** |
| --- | --- | --- | --- | --- |
| ***y w*** | **X^2^= 12.456, df= 2, n= 220, p= 1.8e-03** | **p = 0.14** | **p = 0.041** | **p = 1.3e-03** |
| ***Tak1^1^*** | **X^2^= 32.963, df= 2, n= 258, p= 6.9e-08** | **p = 0.77** | **p = 3.6e-07** | **p = 4.1e-06** |
| ***w upd2∆*** | **X^2^= 66.744, df= 2, n= 237, p= 3.2e-15** | **p = 0.26** | **p = 1.4e-10** | **p = 1.5e-13** |
| ***w;;pdf^01^*** | **X^2^= 13.525, df= 2, n= 237, p= 1.2e-03** | **p = 0.27** | **p = 0.02** | **p = 1e-03** |
| ***dop1R2^MB05108^*** | **X^2^= 15.524, df= 2, n= 90, p= 4.3e-04** | **p = 0.94** | **p = 1.1e-03** | **p = 1.6e-03** |
| ***dopR1^f2676^*** | **X^2^= 44.393, df= 2, n=130, p= 2.3e-10** | **p = 0.81** | **p = 1.4e-08** | **p = 1.1e-08** |
| ***iav^3621^*** | **X^2^= 32.974, df= 2, n= 158, p= 6.9e-08** | **p = 0.36** | **p = 1.6e-05** | **p = 3.8e-07** |
