# Supplementary material for: Infection increases activity via Toll dependent and independent mechanisms in Drosophila melanogaster
Source: PLoS Pathog. 2022 Sep 21;18(9):e1010826. doi: 10.1371/journal.ppat.1010826 (PMC9529128; doi:10.1371/journal.ppat.1010826)
Supplement: S3 Table — Drosophila melanogaster strains used in this study and their sources. (DOCX) [file ppat.1010826.s014.docx]

**S3 Table. Fly strains.**

| Strain | Source |
| --- | --- |
| *w^1118^* | Dionne laboratory stocks |
| Oregon R | Dionne laboratory stocks |
| *imd^10191^* | Dionne laboratory stocks |
| *spzeGFP* | Dionne laboratory stocks |
| *spz*^∆8-1^ | Shoichiro Kurata (Tohoku University) |
| n-syb Gal4 | Gilestro laboratory stocks |
| UAS - AKH-R RNAi | Bloomington Drosophila Stock Center stock 29577 |
| c564 Gal4 | Bloomington Drosophila Stock Center stock 6982 |
| UAS - *spz* RNAi | Vienna Drosophila Resource Center stock 105017 |
| UAS - *myD88* RNAi | Vienna Drosophila Resource Center stock 25399 |
| UAS - *dif* RNAi | Vienna Drosophila Resource Center stock 100537 |
| *y w* | James Jepson (UCL) |
| *tak1^1^* | Bruno Lemaitre (EPFL) |
| *upd2^∆^* | Bruno Lemaitre (EPFL) |
| *pdf^01^* | James Jepson (UCL) |
| *dop1R2^MB05108^* | Bloomington Drosophila Stock Center stock 24743 |
| *dopR1^f2676^* | James Jepson (UCL) |
| *iav^3621^* | Bloomington Drosophila Stock Center stock 24768 |
