# Supplementary material for: Infection increases activity via Toll dependent and independent mechanisms in Drosophila melanogaster
Source: PLoS Pathog. 2022 Sep 21;18(9):e1010826. doi: 10.1371/journal.ppat.1010826 (PMC9529128; doi:10.1371/journal.ppat.1010826)
Supplement: Attachment — Submitted filename: Review PPATHOGENS-D-22-00059.pdf [file ppat.1010826.s016.pdf]

Manuscript Number: PPATHOGENS-D-22-00059

Manuscript Title: Infection increases activity via Toll dependent and independent mechanisms in *Drosophila melanogaster*
Authors: Crystal Vincent, Esteban J. Beckwith, William H. Pearson, Katrin Kierdorf, Giorgio Gilestro, Marc S. Dionne.

Although behavioral changes are among the most apparent effects of infection, the genetic networks that underlie these effects, as well as their overall impacts on both hosts and pathogens remain elusive. In this manuscript, Vincent and colleagues use the model organism *Drosophila melanogaster* to study infection behavior. They discover that the infection with diverse bacterial strains leads to an increase in locomotor activity. Their data further suggest that this response plays a protective role, as there is a positive correlation between increased physical activity and host survival to infection. There is a tight interplay between immunity and metabolism, and the infection with lethal and non-lethal bacteria leads to a reduction in glycogen and triglyceride levels. These metabolic phenotypes are suggestive of a mobilization of energy stores, and activation of AKH/glucagon signaling. AKH is well-known for inducing hyperactivity in starved *Drosophila*, and could therefore play similar roles during infection. The authors, however, ruled out a role for AKH signaling in the hyperactivity induced by the infection with *F. novicida*. Two other obvious candidates are the immune signaling pathways Toll and Imd. The hyperactivity observed upon infection with *F. novicida* is independent of these two pathways. *M. luteus*, however, induces hyperactivity in a Toll-dependent manner, consistent with the fact that this bacterium is an elicitor of the pathway. Altogether, this manuscript demonstrates that the infection with diverse bacteria induces hyperactivity and that this effect could rely on several, unrelated mechanisms. These observations suggest that infection-induced hyperactivity is an important response to the infection with pathogens, and may play important roles in supporting host survival.

This manuscript has previously been submitted to Review Commons, and was accompanied with a "revision plan". The revision plan contains the response of the authors to the comments of the previous reviewers. I however did not have access to the original comments of the first set of reviewers. Some of my comments and suggestions could therefore be redundant with the previous ones, and I apologize if this is the case.

Major comments:

#1 Infection-induced hyperactivity
The authors discovered that the infection with diverse bacterial strains leads to an increase in spontaneous locomotor activity. This goes against the general belief that infection promotes sleep in *Drosophila* (PMID: 30705188, PMID: 24882902, PMID: 20144235). I therefore think that this novel observation needs to be consolidated with more data. For example, I feel it is important to check for infection-induced hyperactivity in several control backgrounds (beyond w^{1118} and *yw* – for example Canton-S, Oregon-R, etc.), and with the different bacterial strains. Is hyperactivity specific to males?

#2 Genetic controls
Some experiments lack genetic controls, or the genetic controls are not depicted in the same figure. This biases the interpretation of some key experiments in the paper.
For example, Figure 5: The genetic controls for these experiments (*C564>/+ and nSyb>/+*) are shown separately, in Figure S7. These controls do not show the hyperactivity induced by *M. luteus* previously seen with w^{1118}: there is no significant difference between the "mock" and "*M. luteus"* treatments (Figure S7, ln 855 and 861). The authors then cannot claim that *spz, myd88,* and *dif RNAi* driven with *C564>* and *nSyb>* suppress hyperactivity in animals infected with *M. luteus* (ln 232-236 and 245-247). The sentence "*Importantly, the genetic control (c564>+) shows the expected increase of activity after bacterial infections (Figure S7)*" is also not correct (ln 236).

Another important point in the paper is that two *spz* mutations suppress *M. luteus*-induced hyperactivity. For the reasons described above, I think it is necessary to add *spz/+* animals as controls. This could

demonstrate clearly that the absence of hyperactivity is indeed caused by the loss of *spz*, and not by variations in the genetic background in these animals.

#3 Bacterial loads

The authors suggest a connection between locomotor activity and pathogen loads. Pathogen loads, however, were not properly assayed (Fig. S5J-K). The authors plated whole-fly homogenates to measure pathogen loads, but this method does not discriminate pathogenic bacteria from commensal (intestinal) bacteria. This explains why in flies infected with *M. luteus*, the authors detected 10^5 CFUs at t=0, while they injected 10^3 CFUs of *M. luteus* (ln. 830). The authors should rather score CFUs in the hemolymph to evaluate *M. luteus* loads. qPCR methods for bacterial detection are mentioned in the methods (ln 374-378), but were not used in the paper.

#4 Food intake experiments

Food intake was not properly assayed (ln 197 – 200).

The authors fed flies with food supplemented with a dye to assay feeding and defecation.

- They sampled whole flies at t=30h and 80h. These samples would give "snapshots", or an instantaneous measure of food content in these flies. Feeding needs to be scored continuously in *Drosophila*, with methods like the CAFE assay to be properly evaluated (PMCID: PMC1899109, PMID: 28362419).
- In parallel, the authors wash the walls of the vials to collect fecal deposits, which are also stained with the alimentary dye. These samples would give a cumulated measure of what has been ingested and defecated, between t=0 and the chosen time point. This method, I feel, would be more accurate than assaying whole fly homogenates at t=30 and t=80h. But if I understood correctly from the methods, the authors pooled the values of the "whole flies" and "vials" samples, instead of analyzing them separately (ln. 428). This therefore skew the interpretation of these experiments.

#5 Data representation / interpretation.

There are discrepancies between how data are displayed in the figures, and how they are interpreted in the text. For example: *«We found that flies infected with F. novicida spent 10% and 9% more time moving than mock injected and uninfected controls, respectively (Figure 1A)"*. Figure 1A, however, shows the "percentage of flies moving over time", not the time spent moving.

Can the authors extrapolate the time spent moving, over a 24h period, from their recordings? This would give the reader a sense of what "hyperactivity" really means.

Similarly, the boxplots in Figures 1A-1D represent the average, over a 3d period, of the percentage of flies that are moving/micro-moving/walking/sleeping. I am not an expert in this type of behavioral assays, but it seems a very convoluted way to represent data. For example, sleep is commonly represented as "minutes of sleep per hour", and not as "the percentage of flies that sleep throughout the day". Could the authors show the time spent moving/walking/sleeping (in minutes) per hour, instead of the "% of flies"?

The legend of Figure 1E (ln 707) is: "*Activity level within the first day of F. novicida infection was positively correlated with survival (Pearson's correlation, 708 r = 0.282; t = 2.96, df = 101, p = 3.9e-03). Data from multiple replicates are shown*". We have no precise information on what is depicted on the figure. What do the dots represent? I assume these are single flies? What is represented on the y-axis? Is this the percentage of time (over a 24h hour period) spent moving? The labels of the y axes in Figure 1E and Figures 1A-D are similar, but could then represent different units/metrics. This just adds confusion. In Figure 1E, could the authors simply plot the total time spent moving (in minutes) at day 1, and the longevity? The same comment applies to Figures 2D and 2E.

#6 Toll signaling and *M. luteus*-induced hyperactivity

An important finding of this study is that Toll seems required for *M. luteus*-induced hyperactivity (Figure 3). Can this observation be recapitulated with another Gram+ bacteria (*S. aureus*)? With another mutant in the Toll pathway? Is Toll activation in the fat body/neurons/hemocytes/other peripheral tissues sufficient to induce hyperactivity in the absence of infection?

#7 Gram negative bacteria, Imd, and hyperactivity

Contrary to what is observed with *M. luteus*, the hyperactivity induced by *F. novicida* infection is independent of Imd signaling. Is this also the case for *L. monocytogenes*? In parallel, do classic, non-intracellular Gram– pathogens (Like *ECC15 and Pseudomonas entomophila*) induce hyperactivity? Is this effect independent of the Imd pathway?

Minor comments:

#8 Validation of the *AKHR RNAi*

The *AKHR* RNAi needs to be validated with RT-qPCR. Does it suppress starvation-induced hyperactivity? Also, AKH doesn't seem required for *F. novicida* to induce hyperactivity, but this doesn't necessarily mean that it does not play a role upon *M. luteus* infection (since the two bacteria employ different mechanisms to promote activity). Is AKH required for *M. luteus*-induced hyperactivity?

#9 Infection-induced hyperactivity in *upd2* and *dopR1* mutants

It seems that the *upd2* and *dopR1* mutants have an exacerbated response to *F. novicida* infection, in terms of physical activity (Figure S4). Proper genetic controls (such as *upd2/+*, etc.) could help determining if these effects are real, and if *upd2* and *dopR1* could regulate infection-induced hyperactivity.

#10 Ln94-95 and 101-103: the sentences are duplicated

#11 Ln143 lacks the reference to Figure 2A

#12 The references to the panels are lacking in the legends of Figure S5 (ln 820-38)
