# Supplementary material for: Infection increases activity via Toll dependent and independent mechanisms in Drosophila melanogaster
Source: PLoS Pathog. 2022 Sep 21;18(9):e1010826. doi: 10.1371/journal.ppat.1010826 (PMC9529128; doi:10.1371/journal.ppat.1010826)
Supplement: Attachment — Submitted filename: Vincent_Beckwith_revision-plan.docx [file ppat.1010826.s015.docx]

**Manuscript number:** RC-2021-01026

**Corresponding author(s):** Marc Dionne

## 1. General Statements [optional]

We want to thank the three anonymous reviewers for their constructive comments. We appreciate the careful and thorough examination of the presented data and the interesting suggestions. These comments will definitely help to improve the work. We understand that, while all the comments are worthy, some of them fall beyond the scope of the present work or represent unnecessary work to sustain our conclusion during this revision.

PLEASE NOTE that due to journal formatting requirements, we have had to renumber the supplementary tables and figures (Fig 1S1 is now S1; 1S2 is now S2; 2S1 is now S3; 2S2 is now S4; 2S3 is now S5; 3S1 is now S6; 5S1 is now S7; Figure 2 supporting table 1 is now Table S1; Figure 2 supporting table 2 is now Table S2). Of course, we cannot edit the reviews.

## 2. Description of the planned revisions

The experiments we plan to do address most of the comments raised by the reviewers. Please, see a *point-by-point* reply below.

- **Reviewer 1 (major comments)**

1. We will add analysis of one more wild type background (Canton S) for one gram-positive and one gram-negative bacteria. In addition, we would like to highlight that many different backgrounds have been employed in the work, as was observed by Reviewer 2.
2. To address this point we will re-evaluate the genotypes in the current Figure 5 with two more gram-positive bacteria. With these experiments we plan to extend the results and support, or not, the generalization regarding the effect of the Gram-positive bacteria.

Regarding the results with Gram-negative bacteria, we agree with the reviewer that a more restricted description of the effect of *F. novicida* will be a better way to treat the presented data. We will change the text accordingly.

3. We will provide comprehensive data and references on the effect of *E. coli* on glycogen and the circulating sugars. These data are already available.
4. We will include an experiment analyzing the effect of heat-killed *F. novicida* and *S. aureus*.

- **Reviewer 1 (minor comments)**

1. We have modified the text in light of the reviewer’s comments, adding clarity and avoiding unnecessary generalizations
2. We reanalyzed the data for days 1 to 4 as suggested and the regression is r= -0.282 with a p‑value = 0.02759. However, it is true that there is a strong effect of the short-lived flies. If we restrict the analysis to days 2 to 4 the regression is not significant. We think the reviewer may be correct that our prior inference was driven exclusively by a small number of outliers, so we have replaced the analysis with this shorter time-period. This change removes the “odd” trend in S. aureus and makes the message clearer: more activity is correlated with increased survival time in two of three lethal infections, while in the last case there is neither a positive nor negative correlation with survival.
3. In the experiments responding to the major comment 2 we will add this control.
4. The measurement was not clearly-expressed in the previous version. We have clarified this in the current version..
5. We have changed the reference accordingly.
6. We have done as the reviewer suggested.
7. We have corrected the typos.

- **Reviewer 2 (major comments)**

1. We will test heat killed bacteria as suggested.
2. We will provide the requested control (n-syb>AKH-R under starvation) and we will employ the same genetic background that was employed in the current Figure 4D.
3. We will provide new experiments to clarify and extend the current results. By reanalyzing Figure 5 genotypes with two more gram-positive bacteria (the same experiments that Rev1 requested in comment 2) we will be able to address the susceptibility of all the genotypes in the same experiments since we will employ lethal bacteria.

- **Reviewer 2 (minor comments)**

1. We have clarified this in the text and the figure—the previous description of this measurement was unclear. The total distance covered is increased, but when we take into account the amount of time the different treatments spend active (normalization of the total distance by the amount of time spend active, that includes walk and micro-movements), all the treatments behave in the same way, i.e., the speed is not altered.
2. The slope of the relationship between “activity during day 1” vs “total activity” is lower than one, and that´s why the activity of day 1, which is not altered, correlated with the lifespan even if the increase is clearer during days 2, 3 and 4. We now provide the “day by day” data (Fig S2) that makes this point clear as requested.
3. We will conduct the suggested experiment
4. We will fix the offset.
5. The c564 driver is not expressed in adult hemocytes in our hands. We will add this information in the manuscript.
6 to 12. We will amend the typos and citations as suggested by the reviewer.

- **Reviewer 3 (major comments)**
- We agree with the reviewer, the metabolic switch that takes place during infection can be critical in this behavioral phenotype, and this is beyond the scope of the present work.
- We will add a complete new data set regarding the role of the toll pathway (also requested by the previous two reviewers). We are confident the new experiments will clarify the results and strength the conclusion.
- **Reviewer 3 (minor comments)**
  1. We have added an explanation of the time-windows. In addition, we will add the long-lasting effects of *M. luteus* as a supplement to figure
  2. We will correct the figure accordingly.
  3. We will correct the figure legend and clarify the nature of the comparisons.
  4. to 13. We have clarified the definitions and concepts in the revised version of the manuscript, added the requested details in the methods, corrected the typos and added the suggested references.

## 3. Description of the revisions that have already been incorporated in the transferred manuscript

## 4. Description of analyses that authors prefer not to carry out

As we mention, we believe that the reviewer’s comments are positive and will improve the work; however, we think that some of the suggested experiments are not necessary to sustain our conclusions. In particular, we are not planning to attend the following concerns.

Reviewer 2 has a general comment on the need of isogenzation of all the genotypes included in the work. We believe that there is no need to redo all the work with new isogenic strains. The reviewer point is that the degree of increase in activity is not always the same in all the experiments and they interpreted this as a lack of reliability of the technique. It is true that the degree of increase in activity due to infection is quantitatively different between experiments, but it is also true that in all of our experiments, we *always* see an increase in activity in flies infected with *Staphylococcus aureus*, *Micrococcus luteus*, *Francisella novicida*, and *Listeria monocytogenes*, and we *never* see an increase in activity in flies infected with *E coli* or *Enterobacter cloacae*. We agree with the reviewer that it is likely that some of the quantitative variability in this effect comes from use of different genetic backgrounds. For instance, in Figure 2 supplement 2 we tested many different mutants that are in different background and all of them show a significant activity increase after infection with *Francisella novicida*, demonstrating that this phenotype is consistent across many different experimental conditions. It is true that we cannot easily compare the magnitudes of increase form one figure to the other, but even if the background were isogenic, we should not statistically compare experiments done separately (and it will not be feasible to include all the genotypes in the same experiment). We believe that it is enough to support our conclusion on a significant increase between genotypes or treatments that run in parallel, and there is no need to compare the magnitude of the increase between different genotypes.

Our interpretation on the richness of using different backgrounds is also supported by the request of reviewer 1 to include different wild type backgrounds in the work.

Rev2, comment 3: We believe that adding an analysis of the relevance of the cellular immune response or the melanization is beyond the scope of the work. Our focus is on the link between bacterial detection and the behavioural phenomenon we document here for the first time; we agree that interactions with effector mechanisms are interesting but they represent an entirely new direction. In response to the same comment, we suggest that *E. faecalis* is difficult to work with in this experimental paradigm because, in our hands, this infection is lethal in under 24 hours or not at all, and the first 24 hours of infection are not amenable to analysis because of confounding effects of anaesthesia and injection.

Rev3, main comment. An additional larger screen for behaviour-modifying genes is beyond the scope of the revision—this screen would take at least a year to do properly and it is unlikely that any gene that emerged from it could simply be published without any further characterization.
