# Supplementary material for: Infection increases activity via Toll dependent and independent mechanisms in Drosophila melanogaster
Source: PLoS Pathog. 2022 Sep 21;18(9):e1010826. doi: 10.1371/journal.ppat.1010826 (PMC9529128; doi:10.1371/journal.ppat.1010826)
Supplement: Attachment — Submitted filename: Response to reviewers_PLOSPathJuly22.docx [file ppat.1010826.s017.docx]

We would like to thank the editors for the opportunity to respond to these reviews. We would also like to thank the reviewers for the very thoughtful reviews received; these encouraged us to look at our data differently and produce a significantly improved contribution to the scientific literature – a great peer review experience. As you will see, we have addressed the editors’ and reviewers’ concerns with a significant amount of new data, as well as editing the manuscript for increased clarity. We believe the manuscript is significantly improved.

1. Repeating the analyses in at least one additional genetic background (reviewer 3).

**We have repeated these experiments using Oregon-R flies, a widely used wild-type stock. These flies recapitulate the activity increase observed throughout the study during both of our focal infections (*F. novicida* and *M. luteus*). These data can be found in Figure S5.**

2. Challenging the effect of dead bacteria (reviewer 2 and 3).

**We have repeated these experiments using heat-killed bacteria of both of our focal bacteria (*F. novicida* and *M. luteus*) and found that HK *F. novicida d*oes not induce increased activity but HK *M. luteus* does*.* These data can be found in Figure S9 and are discussed throughout.**

3. For the key bacterial strains, (*F. novicida* and *M.* luteus), determining whether hyperactivity is induced in both males and females (reviewer 3).

**We have repeated these experiments using female flies and found that females do not exhibit increased activity during either of our focal infections (*F. novicida* and *M. luteus*). These data can be found in Figure S1 and are discussed in lines 385-391 of the discussion.**

4. For the key bacterial strains, determining the minimum OD (reviewer 2).

**Working with the minimal OD would theoretically allow us to observe the effect of bacterial infection on activity, free from any excess or exaggeration of phenotype resulting from an overwhelming number of bacteria. However, this experiment represents a great deal of work, and we think it would be very difficult to interpret if the effect size is related to dose (as seems likely). A further complication is the transient nature of *M luteus* infection: the rapid clearance of these bacteria, especially at low doses, means that the duration, as well as the size, of the effect would rapidly become difficult to measure. In lieu of minimal OD, we have included data on activity during infection with a persistent number of live, non-proliferating *F. novicida*. Though this infection is lethal, we show that activity does not increase in these flies (Figure S10). We also show that infection with a 10-fold lower inoculum of *F. novicida* still results in clear increases in activity (Figure S9).**

5. Working with the minimal OD may be helpful to clarify the claim based on results presented in Fig. 5; as stated by reviewer 3, these data are not convincing. If more significant effects can be obtained in this way, controls corresponding to Fig. S7 should be included in the new Figure 5.

**We repeated these experiments anticipating that an increased sample size would help elucidate whether there is an effect on activity during *M. luteus* infection in the control lines c564>+ and nSyb>+; this new data has made clear that c564>+ flies exhibit increased activity during infection with both *F. novicida* and *M. luteus*. nSyb>+ flies show a significant increase when infected with *F. novicida* but not *M. Luteus*. These findings are now reflected in our interpretations throughout the manuscript, most notably affecting our suggestion that Toll signalling in neurons is required for infection-induced activity during infection with Gram-positive bacteria. We have moved these controls to the main figure (Figure 5) as suggested by the reviewer.**

6. Working at the minimal OD, may also be helpful to clarify the variabilities observed in the mutant analysis (Fig. S4); in particular the apparent stronger effect for upd2 and dop1R1 mutants.

**The reviewers are correct that some of the mutants exhibit an apparent quantitative increase in infection-induced activity. However, as is clear from our data, the size of this effect is already dependent on genetic background, and there is also some inter-experiment variability from non-genetic sources; this is possibly to be expected for a highly complex effect like the one we observe. These issues make us hesitant to overinterpret the effects seen in *upd2* and *dopR1* flies.**

7. Challenging a potential effect of glial cells (reviewer 2).

**Although extremely interesting, exploring the role of this vastly diverse and pervasive group of cells is beyond the scope of the present work.**

8. For ease of reading, modifying the presentation of some results (reviewer 3).

**We appreciate the reviewer’s comment and have adjusted the text and figures to make them less ambiguous. In particular, we changed the y-axis in the ethograms to better describe the way we quantify the activity of the flies during infections. We made parallel changes in the methods section and the figure legends clarifying the way we quantify activity. In brief, we parse the animal behaviour into three categories (walking, micro-moving or quiet) and measure the amount of time that each fly spends in each category, then we express the data as the proportion of a given time period that the animals spend engaged in each type of behaviour. For the ethogram plots, the average activity for each fly is calculated every 30 minutes, and the populational value is presented with the corresponding 95% confidence intervals. For boxplots, we defined different specific time windows (1 day or 3 days, depending on the infection) and can describe the distribution of a certain behaviour within the population in that time window. These general concepts are the standard for the neuroethology field, and the precise method to categorise behaviour was developed by one of the groups involved in the work. The citations to the methodological papers have been added to the text for a full description.**

**Point-by-point response to reviewers’ comments**

**Reviewer #1**

**The reviewer has no concerns for us to address, though we very much appreciate their enthusiasm for our work.**

**Reviewer #2**

- For some of the species, the authors should perform a dose response with different concentrations of bacteria. This should allow us to know if the observed effects are concentration-dependent. It would have been better, I think, to work on the minimum OD that gives an effect. Doses that are higher could mask some effects or show additive effects.

**This excellent suggestion was discussed above, in the response to the editor. In short, we include in the revised version two new experiments to address this question. First, we infected flies with *F. novicida* at a dose 10 times lower, this lower initial dose results in a similar behavioural phenotype (Figure S9). Second, we infected flies with *F. novicida* at the same dose but supplemented the food with the bacteriostatic antibiotic tetracycline, resulting in a prolonged low-dose infection in which no increase in activity was observed (Figure S10).**

- The authors should compare dead and live bacteria as a trigger.

**We now show data for heat-killed *M. luteus* and *F. novicida* (Figure S9).**

- I am surprised by the variability of the effects observed, with some differences being barely statistically significant and others showing a very strong effect. Is there an explanation?

**As described by the reviewer, it is true that some effects are larger than others when considering the magnitude of the response. However, we focused our analysis on the statistical contrasts between groups or treatments evaluated together in the same experiments. While we don´t have a comprehensive explanation for these differences, they may come from different sources like fluctuation in the environmental conditions, different genetic backgrounds, subtle changes in the experimental conditions and/or interindividual variabilities.**

- The authors should further characterize the Spz /Toll axis they identify. First, they only tested the effects of inactivating members of the Toll pathway in neurons. Glial cells are also known to respond to bacteria. Are they necessary here too? Are the Toll pathway members also needed in glial cells and if so, in which ones (perineurial, ensheating glia…?).

- The authors should also remove the Toll receptor itself from neurons and glial cells and test whether this impact the flies response to bacteria.

**Although extremely interesting, exploring the role of this vastly diverse group of cells is beyond the scope of the present work.**

- Similarly, Toll pathway-dependent AMP production following M. luteus activation is mediated by pattern recognition receptors such as PGRP-SA and GNBP-1. Are these involved here? These experiments are important because they will tell us whether a completely different mode of Toll pathway activation is at play here.

**Again, we believe this is beyond the scope of the current work. We hope to be able to establish specific signalling and responding cells and the role of different Toll pathway components in the future.**

- It is known that some fat body cells are present around the head. Can the authors find out if the spz ligand that is required to modulate fly activity after infection is produced from both the body and the fat body of the head?

**We have not dissected the role of specific anatomical areas of fat body; in the past, we have found that region-specific fat body drivers were less specific and/or drove weaker expression than suggested in the literature.**

- Hemocytes have been shown to mediate many interorgan communications in flies. To test whether they are also involved here, the authors should monitor the behavior of infected flies in which hemocytes have been removed.

**Again, we agree that this question is very interesting, and we hope to be able to address it in future work.**

**Reviewer #3**

#1 The authors discovered that the infection with diverse bacterial strains leads to an increase in spontaneous locomotor activity. This goes against the general belief that infection promotes sleep in Drosophila (PMID: 30705188, PMID: 24882902, PMID: 20144235). I therefore think that this novel observation needs to be consolidated with more data. For example, I feel it is important to check for infection-induced hyperactivity in several control backgrounds (beyond w1118 and yw – for example Canton-S, Oregon-R, etc.), and with the different bacterial strains. Is hyperactivity specific to males?

**It is true that there is published evidence that infection promotes sleep. Obviously, our data show an opposing effect. We consider this matter in the discussion; in the end, though, we can only report the effects that we observe. In order to increase the evidence to support our conclusions we have now evaluated the effect of infection with *F. novicida* and *M. luteus* in females; the surprising lack of infection-induced hyperactivity in females may be one cause of this difference between our observations and the literature (Figure S1). In addition, we have included a new wild-type genetic control, Oregon-R (Figure S6). *yw* was tested in the original version and is also presented in Figure S6. We would also like to highlight that, across the work, we tested many different genetic backgrounds (different mutants and transgenic strains) that all support our observation, suggesting that increased activity in male flies during infection is a robust phenomenon.**

#2 Some experiments lack genetic controls, or the genetic controls are not depicted in the same figure. This biases the interpretation of some key experiments in the paper. For example, Figure 5: The genetic controls for these experiments (C564>/+ and nSyb>/+) are shown separately, in Figure S7. These controls do not show the hyperactivity induced by M. luteus previously seen with w1118: there is no significant difference between the “mock” and “M. luteus” treatments

(Figure S7, ln 855 and 861). The authors then cannot claim that spz, myd88, and dif RNAi driven with C564> and nSyb> suppress hyperactivity in animals infected with M. luteus (ln 232-236 and 245-247). The sentence “Importantly, the genetic control (c564>+) shows the expected increase of activity after bacterial infections (Figure S7)” is also not correct (ln 236). Another important point in the paper is that two spz mutations suppress M. luteus-induced hyperactivity. For the reasons described above, I think it is necessary to add spz/+ animals as controls. This could demonstrate clearly that the absence of hyperactivity is indeed caused by the loss of spz, and not by variations in the genetic background in these animals.

**We agree and thank the reviewer for the observation. We have now moved the genetic controls to the main figure (Figure 5). We have also added more experiments and re-evaluated the results and conclusions. These changes are reflected throughout the manuscript.**

#3 The authors suggest a connection between locomotor activity and pathogen loads. Pathogen loads, however, were not properly assayed (Fig. S5J-K). The authors plated whole-fly homogenates to measure pathogen loads, but this method does not discriminate pathogenic bacteria from commensal (intestinal) bacteria. This explains why in flies infected with M. luteus, the authors detected 10^5 CFUs at t=0, while they injected 10^3 CFUs of M. luteus (ln. 830). The authors should rather score CFUs in the hemolymph to evaluate M. luteus loads. qPCR methods for bacterial detection are mentioned in the methods (ln 374-378), but were not used in the paper.

**We would like to apologize for this error. The previous initial inoculum values were based on extrapolation. We have repeated this quantification time course in full (including t=0) and confirmed that throughout the bacterial numbers were similar to those observed in the previous experiments. We are therefore comfortable including the new t=0 data here. We are able to identify *M. luteus* colonies on plates through colony morphology. Furthermore, whole-fly homogenates have never resulted in multiple colony types on our plates, nor do the colonies isolated from *M. luteus* infections appear different from those grown directly from overnight cultures of *M. luteus.* In conclusion, we are confident that our plating protocol is an accurate method for determining the number of injected bacteria in our flies.**

**We realize that we were not explicit in the use of qPCR for bacterial quantification and have now made this clarification in the methods. qPCR was used for *F. novicida* quantifications.**

#4 Food intake was not properly assayed (ln 197 – 200). The authors fed flies with food supplemented with a dye to assay feeding and defecation. They sampled whole flies at t=30h and 80h. These samples would give “snapshots”, or an instantaneous measure of food content in these flies. Feeding needs to be scored continuously in Drosophila, with methods like the CAFE assay to be properly evaluated (PMCID: PMC1899109, PMID: 28362419). In parallel, the authors wash the walls of the vials to collect fecal deposits, which are also stained with the alimentary dye. These samples would give a cumulated measure of what has been ingested and defecated, between t=0 and the chosen time point. This method, I feel, would be more accurate than assaying whole fly homogenates at t=30 and t=80h. But if I understood correctly from the methods, the authors pooled the values of the “whole flies” and “vials” samples, instead of analyzing them separately (ln. 428). This therefore skew the interpretation of these experiments.

**We were possibly unclear on this in the prior version. The approach used is to quantitate total dye present in excreta plus dye present in the fly at the end of the feeding period. It thus captures food eaten and excreted as well as that eaten not long before sample collection. We have not observed the accumulation of dye, that is, once placed on ‘normal’ non-dyed food, no dye is visible throughout the fly’s body. Thus, variation in dye accumulation is not expected to affect our measurement. The sample is centrifuged to exclude absorbance due to opaque insoluble fly-derived particulates and the absorbance is measured at a wavelength at which the fly alone does not show strong absorbance. We think this is the best way of measuring food consumption over long periods using these reagents.**

#5 There are discrepancies between how data are displayed in the figures, and how they are interpreted in the text. For example: «We found that flies infected with F. novicida spent 10% and 9% more time moving than mock injected and uninfected controls, respectively (Figure 1A)”. Figure 1A, however, shows the “percentage of flies moving over time”, not the time spent moving. Can the authors extrapolate the time spent moving, over a 24h period, from their recordings? This would give the reader a sense of what “hyperactivity” really means.

Similarly, the boxplots in Figures 1A-1D represent the average, over a 3d period, of the percentage of flies that are moving/micro-moving/walking/sleeping. I am not an expert in this type of behavioral assays, but it seems a very convoluted way to represent data. For example, sleep is commonly represented as “minutes of sleep per hour”, and not as “the percentage of flies that sleep throughout the day”. Could the authors show the time spent moving/walking/sleeping (in minutes) per hour, instead of the “% of flies”? The legend of Figure 1E (ln 707) is: “Activity level within the first day of F. novicida infection was positively correlated with survival (Pearson’s correlation, 708 r = 0.282; t = 2.96, df = 101, p = 3.9e- 03). Data from multiple replicates are shown”. We have no precise information on what is depicted on the figure. What do the dots represent? I assume these are single flies? What is represented on the yaxis? Is this the percentage of time (over a 24h hour period) spent moving? The labels of the y axes in Figure 1E and Figures 1A-D are similar, but could then represent different units/metrics. This just adds confusion. In Figure 1E, could the authors simply plot the total time spent moving (in minutes) at day 1, and the longevity? The same comment applies to Figures 2D and 2E.

**This issue was dealt with as one of the points specifically put forth by the editor, above. In short, we agree with the reviewer that some aspects were unclear in the previous version of the manuscript. We now express the data as “percentage of time moving” in all the ethograms and boxplots, as well as in the text. To gain clarity, we made explicit in the figure legends that we employ 30 minutes bins to construct the ethogram plots. This is the most common summarizing time employed in the literature. In the case of the boxplots, we selected a time window of analysis based on the characteristics of the different infections (see Methods/Behavioural experiments sections for a description of the criteria).**

**We would like to clarify that, while we show that infections result in more time spent active, we do not describe the flies as hyperactive. This is because the distance the flies cover when they are active doesn’t change with infection (Figure S2D-E), i.e., the infected flies move at the same speed, but for longer periods of time.**

**Regarding main Figures 1E, 2D and 2E, we changed the y-axis to be clearer and consistent with the rest of the figures. We also extended the legend to address the reviewers’ concerns.**

#6 An important finding of this study is that Toll seems required for M. luteus-induced hyperactivity (Figure 3). Can this observation be recapitulated with another Gram+ bacteria (S. aureus)? With another mutant in the Toll pathway? Is Toll activation in the fat body/neurons/hemocytes/other peripheral tissues sufficient to induce hyperactivity in the absence of infection?

**As requested by the reviewer we tested the requirement of the Toll pathway during infections with *S. aureus*. This data set is now added as Figure S7.**

#7 Contrary to what is observed with M. luteus, the hyperactivity induced by F. novicida infection is independent of Imd signaling. Is this also the case for L. monocytogenes? In parallel, do classic, nonintracellular Gram– pathogens (Like ECC15 and Pseudomonas entomophila) induce hyperactivity? Is this effect independent of the Imd pathway?

**We would like to highlight that we have infected flies with the Gram negative, extracellular pathogens *E. coli* and *E. cloacae*, neither of which result in increased activity (Figure S4). We further tested the need for Imd signalling to induce increased activity during *Listeria monocytogenes* infections; these data are presented in Figure S8.**

#8 The AKHR RNAi needs to be validated with RT-qPCR. Does it suppress starvation-induced

hyperactivity? Also, AKH doesn’t seem required for F. novicida to induce hyperactivity, but this

doesn’t necessarily mean that it does not play a role upon M. luteus infection (since the two bacteria employ different mechanisms to promote activity). Is AKH required for M. luteus-induced hyperactivity?

**We have verified that our AKHR RNAi is able to recapitulate the published effect on starvation-induced activity. This is included as Figure S11. We also tested the role of AKHR upon infection with *M. luteus* (Figure 4D).**

#9 It seems that the upd2 and dopR1 mutants have an exacerbated response to F. novicida infection, in terms of physical activity (Figure S4). Proper genetic controls (such as upd2/+, etc.) could help determining if these effects are real, and if upd2 and dopR1 could regulate infection-induced hyperactivity.

**It is true that the magnitude of the increase in some mutants seems larger than in others. However, the goal of these experiments was to test the requirement for the tested genes in the phenotype; it would take much more extensive experimentation to determine the validity of quantitative increases in activity like those that seem apparent in these genotypes. We agree that it would be interesting to know whether the apparent increase seen in these mutants is real and caused by the annotated mutations, but this is out of the scope of the current work.**

#10 Ln143 lacks the reference to Figure 2A

**We fixed this omission.**

#11 The references to the panels are lacking in the legends of Figure S5 (ln 820-38)

**We fixed this omission.**
